# Supplementary material for: From correlation to causation: Estimating effective connectivity from zero-lag covariances of brain signals
Source: PLoS Comput Biol. 2018 Mar 26;14(3):e1006056. doi: 10.1371/journal.pcbi.1006056 (PMC5886625; doi:10.1371/journal.pcbi.1006056)
Supplement: S1 Text — (PDF) [file pcbi.1006056.s001.pdf]

Supporting information

Algorithm 1 Conjugate gradient descent

initialize U, B_0
calculate gradient d and $a \longleftarrow \frac{1}{2}(d - d^*)$
calculate step size δ
$U \longleftarrow \exp(-\delta a)$
while not converged **do**
    $a' \longleftarrow a$
    calculate gradient a
    $\beta \longleftarrow \frac{\langle a, a+a' \rangle}{\langle a', a' \rangle}$
    $g \longleftarrow -a - \beta a'$
    calculate step size δ of g
    $U \longleftarrow \exp(\delta g)$
Linesearch(U, B_0)

Algorithm 2 Line Search with Armijo step size rule

function LINESEARCH$(U_{\text{act}}, d, \alpha)$ ▷ current estimate U_{act}, gradient d, initial-step length α
    $U \longleftarrow exp(-\alpha d)$
    $Q \longleftarrow UU$
    while $\Gamma(U_{\text{act}} B_0) - \Gamma(Q B_0) \geqslant \alpha \langle d, d \rangle$ **do**
        $U \longleftarrow Q$
        $Q \longleftarrow UU$
        $\alpha \longleftarrow 2\alpha$
    while $\Gamma(U_{\text{act}} B_0) - \Gamma(U B_0) \geqslant 0.5\alpha \langle d, d \rangle$ **do**
        $U \longleftarrow exp(-\alpha d)$
        $\alpha \longleftarrow 0.5\alpha$
    return U, α

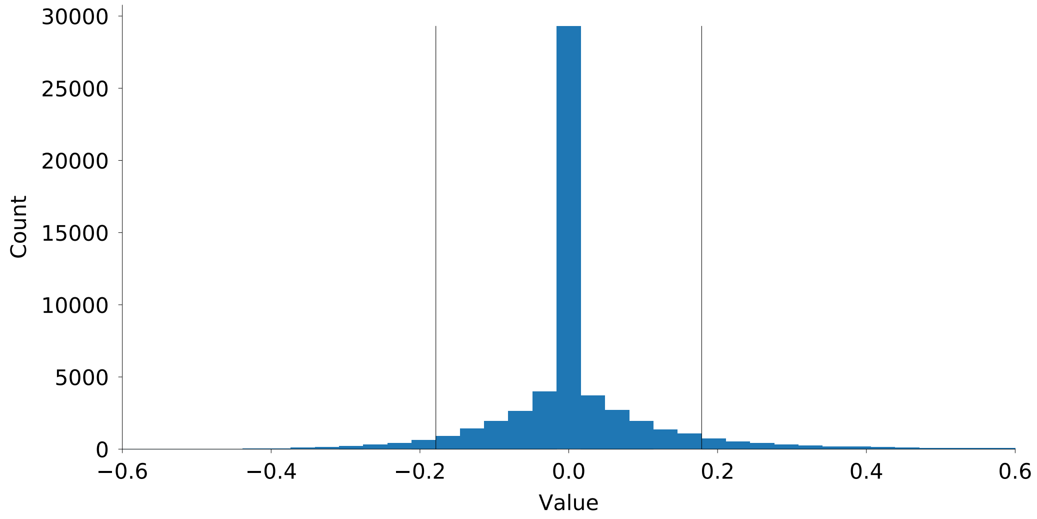

[H]

Figure A. Histogram of estimated connection strengths taken from the reconstructed networks of all seven subjects. The vertical lines show the thresholds for the excitatory and inhibitory connections, respectively. Only a part of the histogram is shown, the actual range of values is between −0.99 and 2.54.

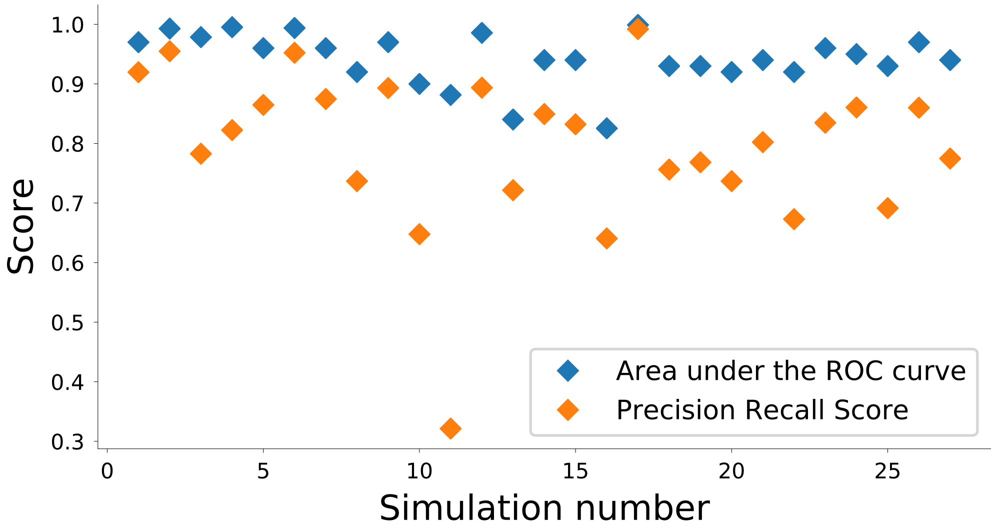

Figure B. Performance of our new inference method on the *NetSim* dataset published by [4]. Other methods have also been tested on these simulated data sets [1, 2, 3]. The x-axis represent the indices of simulated data sets, as in the original publication. The y-axis shows the AUC and PRS of our estimations. We estimated the connectivity for every individual subject and applied a threshold of 50%, the resulting networks were then averaged over all available subjects/trials. Although the networks considered in this paper cover a range of parameters, where we found that our method performs sub-optimally (the networks are generally too small), it still performs reasonably well on these synthetic data. We obtained average values for AUC and PRS of 0.94 and 0.79, respectively.

References

[1] Nie L, Yang X, Matthews PM, Xu ZW, and Guo YK. Inferring functional connectivity in fMRI using minimum partial correlation. International Journal of Automation and Computing, 14:371–385, 2017. doi:10.1007/s11633-017-1084-9

[2] Ryali S, Chen T, Supekar K, Tu T, Kochalka J, Cai W, and Menon V. Multivariate dynamical systems-based estimation of causal brain interactions in fMRI: Group-level validation using benchmark data, neurophysiological models and human connectome project data. Journal of Neuroscience Methods, 268:142–153, 2016. doi:10.1016/j.jneumeth.2016.03.010

[3] Hyvärinen A and Smith SM. Pairwise likelihood ratios for estimation of non-gaussian structural equation models. Journal of Machine Learning Research, 14(1):111–152, 2013.

[4] Smith SM, Miller KL, Salimi-Khorshidi G, Webster M, Beckmann CF, Nichols TE, Ramsey JD, Woolrich MW. Network modelling methods for fMRI. NeuroImage 2011;54;2:875–891. doi:10.1016/j.neuroimage.2010.08.063.

[5] Zaghlool SB and Wyatt CL. Missing data estimation in fMRI dynamic causal modeling. Frontiers in Neuroscience, 8:191, 2014. doi:10.3389/fnins.2014.00191
